# Short Body Height and Pre-pregnancy Overweight for Increased Risk of Gestational Diabetes Mellitus: A Population-Based Cohort Study

**DOI:** 10.3389/fendo.2018.00349

**Published:** 2018-06-26

**Authors:** Jing Li, Peng Wang, Cuiping Zhang, Junhong Leng, Nan Li, Leishen Wang, Wei Li, Huikun Liu, Zhijie Yu, Gang Hu, Juliana C. N. Chan, Xilin Yang

**Affiliations:** ^1^Department of Epidemiology and Biostatistics, School of Public Health & National Demonstration Center for Experimental Preventive Medicine Education, Tianjin Medical University, Tianjin, China; ^2^Tianjin Women and Children's Health Center, Tianjin, China; ^3^Population Cancer Research Program and Department of Pediatrics, Dalhousie University, Halifax, NS, Canada; ^4^Chronic Disease Epidemiology Laboratory, Pennington Biomedical Research Center, Baton Rouge, LA, United States; ^5^Department of Medicine and Therapeutics Hong Kong Institute of Diabetes and Obesity and The Chinese University of Hong Kong-Prince of Wales Hospital-International Diabetes Federation Centre of Education, Hong Kong, Hong Kong

**Keywords:** gestational diabetes mellitus, body height, pre-pregnancy overweight, synergistic effect, Chinese women

## Abstract

**Background:** Short height is associated with gestational diabetes mellitus (GDM) but the underlying mechanism remains unknown. This study aims to explore whether short height has a synergistic effect with pre-pregnancy overweight/obesity and undue weight gain on the risk of GDM.

**Methods:** We recruited 19,962 singleton pregnant women from their first antenatal care visit in urban Tianjin, China, between October 2010 to August 2012. At 24–28 weeks of gestation, women underwent a 50-g 1-h glucose challenge test (GCT) followed by a 75-g 2-h oral glucose tolerance test (OGTT) if the GCT result was ≥7.8 mmol/L. GDM was defined by the International Association of Diabetes and Pregnancy Study Group's cut-points. Univariable and multivariable logistic regression analyses were performed to obtain odds ratios (ORs) and 95% confidence intervals (CIs). Restricted cubic spline (RCS) analysis nested in the logistic regression analysis was used to identify a cutoff point of height for GDM. Additive interaction was used to test interactions between short height, pregnancy overweight/obesity and undue weight gain.

**Results:** A total of 1,517 (or 7.6%) women developed GDM. The risk of GDM increased rapidly with a decreasing height from 158 cm and downwards. Using height ≥158 cm as the reference group, women with < 158 cm of height were at increased GDM risk (adjusted OR: 1.44, 95%CI: 1.18–1.75). Maternal overweight/obesity at the first antenatal care visit greatly enhanced the OR of short height for GDM (adjusted OR: 3.78, 95%CI: 2.84–5.03) with significant additive interaction (*P* < 0.05). However, the interaction between short height and undue weight gain was non-significant (*P* > 0.05).

**Conclusions:** In Chinese pregnant women in urban Tianjin, height < 158 cm had a synergistic effect with pre-pregnancy overweight/obesity on the risk of GDM.

## Introduction

In the past decades, the prevalence of gestational diabetes mellitus (GDM) has been increasing all over the world including China (1). For example, the prevalence of GDM in Tianjin, China, had increased from 2.3% in 1999 to 8.1% in the period from 2010 to 2012 (2, 3). It is well-established that GDM is associated with adverse pregnancy outcomes in the short run and also has adverse health impacts on both women with prior GDM and their offspring in the long run. In this regard, women with GDM are at higher risk of hypertension, preeclampsia, infection and cesarean delivery and delivery of a macrosomic infant. The neonates from mothers with GDM are at high risk of fetal hypoglycaemia, hyperinsulinemia, hypocalcaemia, and respiratory distress (4, 5). Our meta-analysis showed that early lifestyle prevention within 15th gestational week was able to reduce the occurrence of GDM (6). More importantly, the efficacy was not limited to pre-pregnancy obese women but extended to women at high risk of GDM due to presence of other GDM risk factors (7). For possible intervention in the early pregnancy, it is essential to identify women with high risk of GDM. In addition to traditional risk factors, several studies reported that there was a negative association between body height and glucose intolerance in non-pregnant and non-diabetes subjects (8) and the association existed in both lean and obese subjects (9). Short height at birth has been reported to have long-term deleterious impacts on metabolism (10) and is associated with increased risk of GDM in Caucasians (11) and Asian women (2, 12). It is also yet to establish a cutoff point of short height to identify women at high risk of GDM in Chinese population.

Biological links between short height and increased risk of GDM are complex and still unclear. Adult height is a cumulative result of nutritional environment and genetic factors over the growing period. Presumably, under-nutrition during the key periods related with short height may cause catch-up growth, especially through the acquisition of “thrifty phenotype” in adverse intrauterine milieu. Catch-up growth is recognized as a risk factor for glucose intolerance in later life due to undue weight gain and obesity increasing insulin resistance (13, 14). It is also possible that nutritional deprivation leading to short height in childhood and early-life impairs the development of beta cells and their function (15, 16). Similar to type 2 diabetes, GDM is characterized by decreased beta cell function and increased insulin resistance, the latter either stemming from persisting insulin resistance from pre-pregnancy or insulin resistance induced by pregnancy or both. If decreased beta cell function plays a dominant role in the association between short height and GDM, we can assume that short height has a synergistic effect with pre-pregnancy obesity (i.e., pre-pregnancy insulin resistance) and/or undue weight gain (i.e., pregnancy-induced insulin resistance) on the risk of GDM.

Using an established population-based cohort of Chinese pregnant women in Tianjin, China, this study aims (1) to define a cutoff point of short height for the risk of GDM and (2) to test the hypothesis that short height and pre-pregnancy overweight/obesity, or short height and undue weight gain during pregnancy have a synergistic effect toward increasing the risk of GDM in Chinese pregnant women.

## Materials and methods

### Study population and settings

Tianjin, the fourth largest city of China, is located at 137 kms southeast of Beijing and consists of six central urban districts, one new urban district, four suburban districts, and five counties. At the end of 2012, the city of Tianjin had over 14 million residents, of which about 4.3 million lived in the six central urban districts where the study was conducted.

The antenatal care in the six urban districts was shared by three levels of antenatal care institutions, i.e., primary, secondary and tertiary care hospitals. The 3-tier prenatal care system was consisted of 65 primary hospitals (tier one), six district-level Women and Children's Health Center (WCHC) and other secondary obstetric hospitals (tier two), and a city-level Tianjin WCHC and other tertiary care hospitals (tier three). Tianjin WCHC played a key role in coordinating antenatal care by these medical institutions. All pregnant women were initially registered with a primary hospital and received antenatal care there until 32nd gestational week and then referred to one of secondary or tertiary care hospital of their choice. In 1998, our team established a universal screening and management system for GDM within the 3-tiered antenatal care network (2).

From October 2010 to August 2012, we set up a cohort of pregnant women with data collected from their first antenatal care visit till delivery and early postpartum period. During this period, 22,069 singleton pregnant women registered with a primary care hospital. We sequentially excluded 1233 women who did not undergo the GCT, 870 women with positive GCT but did not undergo the OGTT and 4 women with missing information on height. A total of 19,962 pregnant women were included in the final analysis. The ethics clearance was obtained from the Ethics Committee for Clinical Research of Tianjin WCHC. The study was carried out in accordance with the Declaration of Helsinki and written informed consent was obtained from these women before data collection. In 2009, the 3-tier prenatal care system set up a computerized Maternal and Child Health Information System in Tianjin to share data of pregnant women by care-givers at different levels.

### Screening and diagnosis of GDM

GDM was identified using a two-step procedure. All the pregnant women were offered a 50-gram 1-h GCT in non-fasting status in 24th to 28th weeks of gestation at a primary care hospital. Those who had plasma glucose (PG) reading ≥7.8 mmol/L were referred to the GDM clinic located within TWCHC for a standard 75-gram 2-h OGTT. The OGTT was performed after an overnight fasting of at least 8 h. The OGTT results were interpreted according to the International Association for Diabetes in Pregnancy Study Group's (IADPSG) criteria, i.e., having met any one of the cutoff points: fasting PG ≥ 5.1 mmol/L, 1-h PG ≥ 10.0 mmol/L or 2-h PG ≥ 8.5 mmol/L (17).

### Data collection and definitions

We collected demographic information, lifestyle, pregnancy-related medical conditions from these women at their first antenatal care visits and 24–28 weeks of pregnancy, such as age, parity and family history of diabetes, smoking, and drinking habits in a longitudinal manner, using a set of specially designed questionnaires (3, 18). The pregnancy outcomes including gender of infants were retrieved from the Maternal and Child Health Information System.

### Measurements and clinical definitions

Anthropometric and clinical measurements of all subjects were measured by uniformly-trained staff members with a standardized protocol and tools. Height was measured to the nearest 0.5 cm and weight was measured to the nearest 0.1 kg. Height and weight were measured in women wearing light closing and without shoes at first antenatal care visit and weight was re-measured at GCT time. Body weight at first antenatal care visit was recorded as pre-pregnancy weight. Difference in body weight from first antenatal care visit to GCT time was estimated as gestational weight gain. Undue weight gain defined as ≥75th percentile (i.e., 0.37 kg per week). Body mass index (BMI) was calculated to estimate adiposity as the ratio of weight in kilograms to height squared in meters and categorized for overweight and obesity according to Chinese adults' criteria (19), i.e., underweight: BMI < 18.5 kg/m^2^; normal body weight: BMI at 18.5–23.9 kg/m^2^; overweight: BMI at 24–27.9 kg/m^2^, obesity: BMI ≥ 28 kg/m^2^. Sitting blood pressure (BP) was measured from right arm after at least 10 min rest when then underwent GCT.

Maternal age was calculated as the period in years from the date of birth to the date of first antenatal care visit. Educational attainment was divided into two categories: junior college or below, and tertiary education or above. Family history of diabetes was defined as having any first-degree relatives with diabetes. Habitual smoker was defined as continuously smoking one or more cigarettes per day for at least 6 months before or during pregnancy. Habitual drinker was defined as drinking occasionally or once or more per week before or during pregnancy.

### Statistical analysis

All analyses were performed using the Statistical Analysis System (Release 9.2) (SAS Institute Inc., Cary, USA) and all data were expressed as mean ± standard deviation (SD) or median (interquartile range, IQR) where appropriate. Student's *t*-test or Wilcoxon two-sample test was used to compare means (or median) of continuous variables. Chi-squared test (or Fisher exact test where appropriate) was used to compare categorical variables between the GDM group and the non-GDM group. Binary logistic regressions were performed to obtain odds ratios (OR) and 95% confidence intervals (CI) of height and other variables under study for GDM in univariable and multivariable analyses. In the multivariable analysis, we adjusted for traditional GDM risk factors, including age, habitual smoker, alcohol drinker, Han-ethnicity, parity, systolic/diastolic BP at GCT, education attainment, family history of diabetes on the first-degree relations as well as infant gender.

Restricted cubic spline (RCS) is piecewise cubic polynomials connected across different intervals of a continuous variable, which can fit sharply curving shapes (20). We used to employ this method in numbers of our previous studies to identify cutoff points of lipids for cancer in type 2 diabetes (21) and alanine aminotransferase for GDM (22). In this study, we used RCS nested in logistic regression analysis to examine the full range association between height and the risk of GDM and to define cutoff points of height for GDM if any. Briefly, we chose 4 knots at quintiles 0.05, 0.35, 0.65, and 0.95 as suggested by Harrell (20). ORs between two heights can be estimated by EXP (Y_2_–Y_1_), where Y_2_ and Y_1_ were the values of RCS functions at heights 2 and 1. As before, a cutoff point was selected if the risk of GDM rapidly increased since that point by visual checking of the curve's shape. Further confirmation logistic regression analysis was performed by stratifying height into a binary variable at a selected cutoff point.

Synergistic effects between short height and pre-pregnancy overweight and undue weight gain from the first antenatal care visit to GCT time were estimated using additive interaction (23). Three measures, i.e., relative excess risk due to interaction (RERI), attributable proportion due to interaction (AP), and synergy index (SI), were used to estimate additive interaction. A significant RERI > 0, AP > 0, or SI > 1 indicates an additive interaction or synergistic effect between short stature and overweight/undue weight gain for GDM. A calculator was available at http://epinet.se/res/xls/epinetcalculation.xls. (23) Because age is among the strongest risk factors for GDM, we also performed additional analysis to test additive interaction between short weight and old age (i.e., ≥30 years).

Additional analysis using analysis of covariance (ANCOVA) was performed to compare adjusted means of BMI and weight gain between women with short height and their counterparts without short height. Sensitivity analysis was also performed to check consistency of the results after exclusion of 1,209 women who registered after 14th gestational week in the main analysis.

## Results

The 19,962 pregnant women had a mean age of 28.5 (*SD*: 2.9) years, a mean height of 163.2 (*SD*: 4.7) cm, a mean body weight of 59.5 (*SD*: 9.8) kg and a mean BMI of 22.3 (*SD*: 3.4) kg/m^2^. These women gained a mean body weight of 0.29 (*SD*: 0.2) kg per week from registration to the GCT time. Of them, 7.6% (*n* = 1,517) developed GDM. Women with GDM had an older age, shorter height, heavier body weight, higher pre-pregnancy BMI and higher PG at GCT. They were also more likely to be multiparous, to give birth to a male infant and to have family history of diabetes in first degree relatives and higher BPs (Table 1).

**Table 1:** Clinical and biochemical characteristics of subjects according to occurrence of gestational diabetes mellitus diagnosed by the IADPSG's criteria.

	**Non-GDM**	**GDM**	***P*-value**
N	18,445	1,517	
**AT REGISTRATION WITH PREGNANCY**			
Age, year	28.4 ± 2.9	29.5 ± 3.3	< 0.001^*^
Age group, year			
< 25	1,845 (10.0%)	72 (4.7%)	< 0.001^**^
≥25~ < 30	1,2176 (66.0%)	907 (59.8%)	
≥30	4,424 (24.0%)	538 (35.5%)	
Height. cm	163.2 ± 4.7	162.7 ± 4.8	0.001^*^
Height group < 158 cm	1,530 (8.3%)	162 (10.7%)	
Body weight, kg	59.1 ± 9.5	64.1 ± 11.5	< 0.001^*^
Pre-pregnancy BMI, kg/m^2^	22.2 ± 3.3	24.2 ± 3.9	< 0.001^*^
**BMI GROUP, kg/m**^2^			
< 18.5	1,892 (10.3%)	58 (3.8%)	< 0.001^**^
≥18.5– < 24	12,044 (65.3%)	755 (49.8%)	
≥24– < 28	3,423 (18.6%)	483 (31.8%)	
≥28	1,086 (5.9%)	221 (14.6%)	
Nationality			0.016^**^
Han	17,591 (95.4%)	1,467 (96.7%)	
Parity			0.008^**^
≥1	686 (3.7%)	77 (5.1%)	
Education attainment			0.845^**^
Junior college and below	8,404 (45.6%)	688 (45.4%)	
Tertiary education	10,041 (54.4%)	829 (54.7%)	
Family history of diabetes in first degree relatives	1,381 (8.2%)	206 (15.8%)	< 0.001^**^
Gestation week at registration	10.9 ± 2.4	10.8 ± 2.3	0.109^*^
**AT GCT**			
Plasma glucose	6.3 ± 1.3	9.4 ± 1.5	< 0.001^*^
Weight gain from first antenatal care visit to GCT, kg	7.4 ± 7.3	7.3 ± 3.6	0.848 ^*^
Weight gain per week, kg/wk	0.3 ± 0.2	0.3 ± 0.1	0.760^*^
Weight gain group, kg/wk			0.842^**^
More (>0.37)	4,358 (23.6%)	355 (23.4%)	
Gestational weeks at GCT, week	25.2 ± 2.5	25.3 ± 1.8	0.181^*^
Systolic BP at GCT, mmHg	106.2 ± 10.5	109.6 ± 10.	< 0.001^*^
Diastolic BP at GCT, mmHg	68.2 ± 7.4	70.3 ± 7.8	< 0.001^*^
Smoker^†^	176 (1.0%)	19 (1.3%)	0.256^**^
Drinker^‡^	5,620 (30.5%)	459 (30.3%)	0.863^**^
**AT DELIVERY**			
Male infant gender	9,398 (51.8%)	846 (56.4%)	0.003^**^

*BMI, body mass index; GCT, glucose challenge test; BP, blood pressure; Data are reported in mean ± SD or number (%)*.

* *Derived from Student's t- test*.

** *Derived from Chi-square test or Fisher's Exact Test*.

† *Defined as smoking one or more cigarette per day before or during pregnancy*.

‡ *Defined as drinking occasionally or once or more per week before or during pregnancy*.

**Figure 1:**
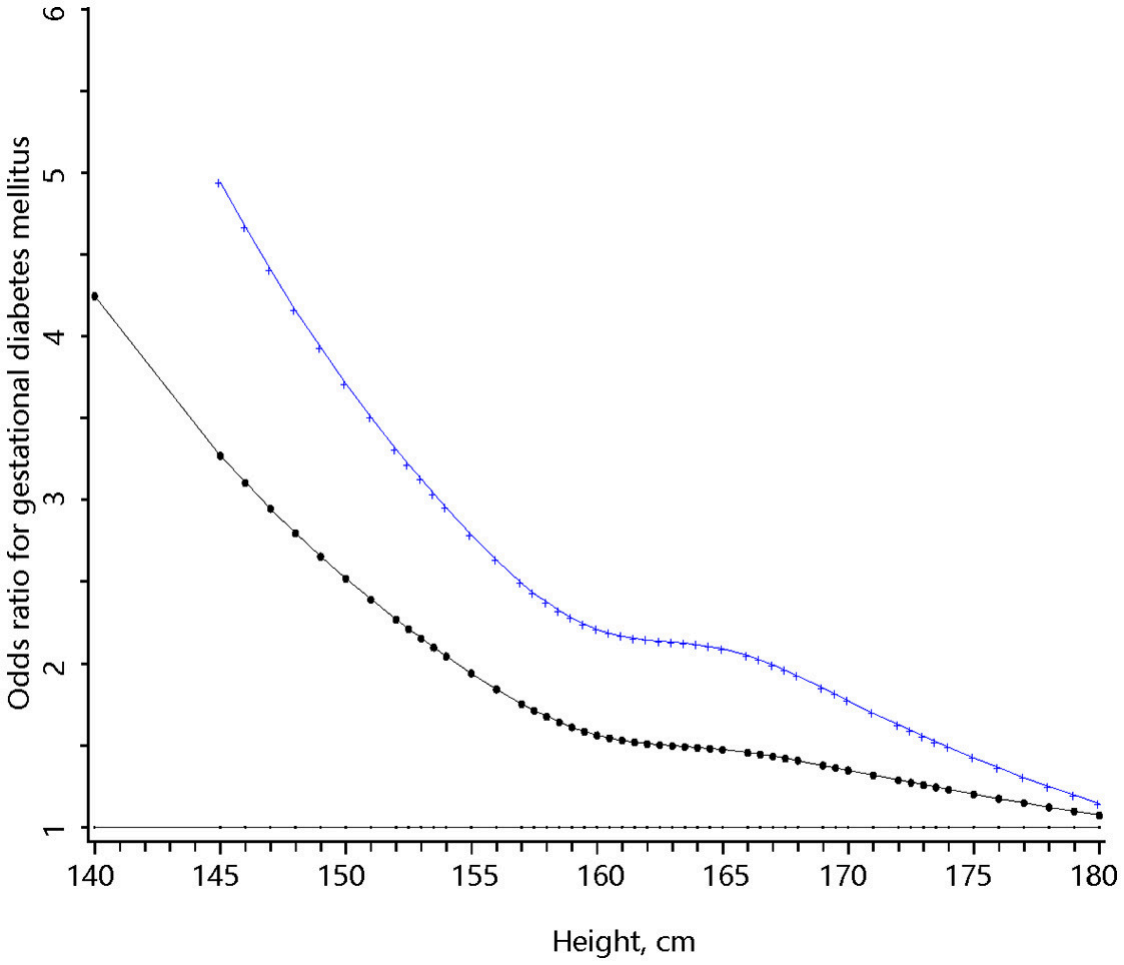
The full range association between body height and the risk of gestational diabetes mellitus. The bottom (dotted) curve was derived from univariable analysis and the upper (cross) curve was derived from multivariable analysis that adjusted for pre-pregnancy body mass index, weight gain per week from first antenatal care visit to glucose challenge test (GCT), age, habitual smoker and drinker, Han-ethnicity, parity, systolic, and diastolic blood pressure at GCT, education, family history of diabetes, and baby gender.

Height was inversely associated with the risk of GDM in multivariable analysis. The OR of GDM increased with decreasing height down to 158 cm and the risk of GDM rapidly increased in a linear manner with decreasing height from 158 cm downwards (Figure 1). If 158 cm was used as the cutoff point to define short height, 8.5% (*n* = 1,692) of the women had short height and 9.6% (n = 162) of the short women developed GDM. Women with short height were at higher risks of GDM in univariable analysis and multivariable analysis (Table 2). Compared to normal weight, overweight, or obesity (BMI ≥ 24 kg/m^2^) was also associated with increased risk of GDM. In multivariable analysis, women with short height had similar pre-pregnancy BMI but gained less as compared with women with taller height (Appendix Table 1 in Supplementary Material).

**Table 2:** Odds ratios (ORs) of short height and pre-pregnancy BMI for the risk of gestational diabetes mellitus.

	***N* (%)**	**Odds ratio**	**95% confidence intervals**	***P*-value**
**INDEPENDENT MODELS**				
**Univariable analysis**^†^
Height < vs. ≥158 cm	162 (10.7%)	1.32	1.11–1.57	0.001
BMI ≥ 24 vs. < 24 kg/m^2^	704 (46.4%)	2.68	2.41–2.98	< 0.001
Multivariable analysis^‡^
Height < vs. ≥ 158 cm	126 (11.2%)	1.44	1.18–1.75	< 0.001
BMI ≥ 24 vs. < 24 kg/m^2^	527 (46.9%)	2.33	2.04–2.65	< 0.001
**INDEPENDENT MODELS IN THE SUBGROUPS**				
**Univariable analysis among women with BMI** ≥ **24 kg/m**^2^^†^
Height < vs. ≥ 158 cm	83 (11.8%)	1.46	1.13–1.87	0.004
**Multivariable analysis among women with BMI** ≥ **24 kg/m**^2^^‡^
Height < vs. ≥ 158 cm	66 (12.5%)	1.68	1.26–2.24	< 0.001
**Univariable analysis among women with BMI**<**24 kg/m**^2^^†^
Height < vs. ≥ 158 cm	79 (9.7%)	1.20	0.94–1.52	0.144
**Multivariable analysis among women with BMI**<**24 kg/m**^2^^‡^
Height < vs. ≥ 158 cm	60 (10.1%)	1.27	0.96–1.67	0.096
**ADDITIVE INTERACTION MODELS AMONG THE WHOLE COHORT**				
**Univariable analysis**^†^
Height < 158 cm and BMI ≥ 24 kg/m^2^	83 (5.5%)	3.82	2.97–4.89	< 0.001
Height < 158 cm and BMI < 24 kg/m^2^	79 (5.2%)	1.20	0.94–1.52	0.144
Height ≥ 158 cm and BMI ≥ 24 kg/m^2^	621 (40.9%)	2.62	2.34–2.93	< 0.001
Height ≥ 158 cm and BMI < 24 kg/m^2^	734 (48.4%)	1	Reference	
**Multivariable analysis**^‡^
Height < 158 cm and BMI ≥ 24 kg/m^2^	66 (5.9%)	3.78	2.84–5.03	< 0.001
Height < 158 cm and BMI < 24 kg/m^2^	60 (5.3%)	1.26	0.96–1.67	0.097
Height ≥ 158 cm and BMI ≥ 24 kg/m^2^	461 (41.0%)	2.26	1.97–2.59	< 0.001
Height ≥158 cm and BMI < 24 kg/m^2^	537 (47.8%)	1	Reference	

*BMI, body mass index; GCT, glucose challenge test; SBP/DBP, systolic/diastolic blood pressure*.

† *Not adjusted for any other variables*.

‡ *The variables adjusted in the multivariable analysis included weight gain per week from first antenatal care visit to GCT, age, habitual smoker and drinker, Han-ethnicity, parity, SBP, and DBP at GCT, education, family history of diabetes, baby gender, in addition to the variables listed in the model*.

In the subgroup analysis, short height was associated with increased risk of GDM among women with pre-pregnancy BMI ≥24 kg/m^2^ in univariable analysis (OR: 1.46; 95%CI: 1.13–1.87) and multivariable analysis (OR: 1.68; 95%CI: 1.26–2.24). On the other hand, the OR of short height for GDM was not significant among women with pre-pregnancy BMI < 24 kg/m^2^ in multivariable analysis (OR: 1.27; 95%CI: 0.96–1.67). If height ≥158 cm and BMI < 24 kg/m^2^ were used as the reference, BMI ≥24 kg/m^2^ alone but not height < 158 cm alone was associated with increased risk of GDM in multivariable analysis. Co-presence of both risk factors greatly increased the OR further to 3.78 (95%CI: 2.84–5.03). All the three additive interaction measures are significant (AP: 0.33, 95%CI: 0.12–0.54; RERI: 1.26, 95%CI: 0.16–2.35 and SI: 1.83, 95%CI: 1.15–2.89) (Table 3).

**Table 3:** Additive interaction between height < 158 cm and BMI ≥ 24 kg/m^2^ for the risk of gestational diabetes mellitus.

**Measures of additive interaction**	**Estimate**	**95% confidence intervals**
**UNIVARIABLE ANALYSIS**^†^		
RERI	1.00	0.03–1.97
AP	0.26	0.06–0.46
SI	1.55	1.06–2.27
**MULTIVARIABLE ANALYSIS**^‡^		
RERI	1.26	0.16–2.35
AP	0.33	0.12–0.54
SI	1.83	1.15–2.89

*BMI, body mass index; AP, attributable proportion due to interaction; RERI, relative excess risk due to interaction; SI, synergy index; Significant RERI > 0, AP>0 or SI>1 indicates a significant additive interaction*.

† *Not adjusted for any other variables*.

‡ *The variables adjusted are the same as those in Table 2*.

If the 75th percentile of weight gain from registration to GCT time was used to define undue weight gain, short height was associated with increased risk of GDM among women with undue weight gain and also among women without undue weight gain. However, all the additive interaction measures between short height and undue weight gain were not significant (Appendix Tables 2, 3 in Supplementary Material).

Short height was also associated with increased risk of GDM in women aged < 30 years but not in women aged ≥30 years. However, the additive interaction between short height and ≥30 years of age was not significant (Appendix Tables 4, 5 in Supplementary Material).

After exclusion of women who registered after the 14th gestational week, the additive interaction between short height and overweight/obesity remained significant in multivariable analysis (AP: 0.28, 95%CI: 0.04–0.51; RERI: 1.01, 95%CI: −0.10 to 2.11; SI: 1.62, 95%CI: 1.01–2.60) (Appendix Tables 6, 7 in Supplementary Material).

## Discussion

This study found that 158 cm was a cutoff point to define short height for GDM in Chinese pregnant women, and height below the cutoff point was associated with markedly increased risk of GDM, and the effect was limited to women with pre-pregnancy BMI ≥ 24 kg/m^2^.

Overweight/obesity and undue weight gain during pregnancy are well-established risk factors for GDM. Consistent epidemiological data also suggested that short height was associated with increased risk of GDM in pregnant women as well as abnormal carbohydrate metabolism in the general population. For example, several studies reported that height was inversely correlated with abnormal glucose tolerance and insulin resistance in non-pregnant subjects without diabetes (8, 24). In one study of 9,471 pregnant women in Tianjin, China (2), we reported that a centimeter increase in height was associated with 4% decrease in the risk of GDM. A Korean study (*n* = 9,005)(12) reported that women in the shortest quartile (≤ 157 cm) were two times more likely to develop GDM than women in the highest quartile (≥163 cm). Similarly, a smaller Greek study (*n* = 2,772) (9) reported an increased risk for GDM in women with a stature in the lowest quartile (< 159 cm). In Brazil, short stature was also associated with GDM risk, with women who had a short stature (≤ 151 cm) having a 60% increase in the odds of GDM (25). Using a more sophisticated method, we further refined the cutoff point of short height for the risk of GDM in Chinese pregnant women and found that women with height < 158 cm had a 1.44-fold increased risk of GDM compared with women who were taller. More importantly, our study generated novel findings that the effect of short height on GDM was limited to overweight/obese women.

GDM may develop due to beta cell dysfunction, high insulin resistance including that persisting from pre-pregnancy and undue insulin resistance induced by pregnancy, or both. It is presumable that pre-pregnancy overweight/obesity is associated with insulin resistance before pregnancy while pregnancy-induced insulin resistance is associated with undue weight gain. Increased pre-pregnancy insulin resistance and undue pregnancy-induced insulin resistance may exert extra burden on the subclinical beta cell function, i.e., increased insulin resistance and beta cell dysfunction having a synergistic effect on GDM. In this connection, short height maybe, presumably, is associated with impaired development of beta cell function or increased insulin resistance, the latter being termed as growth catch-up insulin resistance (16, 26). In our analysis, short height was not associated with higher pre-pregnancy BMI and the association between short stature and GDM was independent of pre-pregnancy BMI. This observation supports the hypothesis that co-presence of impaired beta cell function and insulin resistance greatly predisposes women to GDM but does not support a role of growth catch-up in the association between short height and GDM. In a word, our findings are consistent with the thrifty phenotype hypothesis that the adaptative alterations protecting these women from undernourishment during their early development could have led them to short body height and may also lead to glucose intolerance (27, 28). It is also possible that a genetically determined insulin effect could lead to both failure to grow and diabetes (29, 30). Therefore, the short stature may be a result of thrifty genotype (31), which might have contributed to predisposition of the women to GDM.

It is possible that the observed synergistic effect of short height and overweight/obesity may also work for type 2 diabetes. Dutch Famine study revealed that pregnant women exposed to famine in early pregnancy increased the risk of offspring to develop metabolic diseases in adulthood due to epigenetic modifications (32). Therefore, our findings suggest that it is worthwhile to investigate a possible role of epigenetic modification for the increased risk of GDM among women with short height or both short height and overweight.

Our study has strong public health implications. First, the prevalence of GDM has been increasing globally. Although lifestyle intervention in early pregnancy may reduce the risk of GDM, such intervention is, at best, able to reduce 22% risk of GDM (6) and the residue risk of GDM remains quite high. Further understanding of the etiology of GDM is urgently needed. In this regard, our findings may help better understand GDM. Second, we defined a novel risk marker for GDM, i.e., co-presence of short height and overweight/obesity. This risk marker may be useful to identify those women at high risk of GDM at early pregnancy for possible intervention that had been shown to reduce the risk of GDM (6). It is also noticed that the predictive power of height is not large (27, 33). However, women with both height < 158 cm and overweight/obesity were at particular high risk of GDM and they accounted for 33% of the total GDM cases in the population (i.e., AP = 0.33). Although height is an unmodifiable risk marker, overweight prior to pregnancy is modifiable. Removal of pre-pregnancy overweight in the high-risk group is expected to reduce the excess risk of GDM and can greatly contribute to control of GDM in our population.

Our study had several limitations. First, a two-step procedure in our antenatal care system was used to detect GDM, which might lead to missing of some GDM cases. Second, some women were excluded due to failure to turn up for GCT and/or OGTT. Compared with the women included in the analysis, the excluded women were older and had higher BMI though having a similar height (data not shown). This, the observed effect sizes might underestimate the true effect sizes of risk factors under investigation for GDM. Third, weight gain was calculated as that from registration to GCT time, not that from pre-pregnancy to the GCT time. Fourth, we found body height was inversely associated with increased risk of GDM consisted with earlier studies and the cutoff point of short height (< 158 cm) defined in our cohort was similar with the mean height (157.7 cm) among Asians with GDM in a meta-analysis with large cohorts (27), but given the heterogeneous populations with various ethnicities in China, the cutoff point of height for increased risk of GDM need to be tested in other Chinese populations.

## Conclusion

We found that short height defined as < 158 cm was associated with markedly increased risk of GDM, and the effect was limited to women who were overweight/obese before pregnancy in Chinese population. If further replicated in other cohorts, co-presence of short height and overweight/obesity before pregnancy may be a useful risk marker for identification of women at high risk of GDM who may benefit most from lifestyle intervention before pregnancy. Further research into a possible role of epigenetic modifications in the association between short height and GDM is warranted.

## Author contributions

XY conceived and designed the study. JL analyzed the data and wrote the first draft. PW, CZ, JhL, NL, LW, WL, and HL collected the data. CZ, JhL, NL, LW, WL, and HL collected and assembly the data, gave critical comments on the manuscript. ZY, GH, and JC gave critical comments and edited the manuscripts. All authors read and approved the final manuscript.

### Conflict of interest statement

The authors declare that the research was conducted in the absence of any commercial or financial relationships that could be construed as a potential conflict of interest.

## Abbreviations

AP: attributable proportion due to interaction
BMI: body mass index
BP: blood pressure
CI: confidence intervals
GCT: glucose challenge test
GDM: Gestational diabetes mellitus
IADPSG: International Association of Diabetes and Pregnancy Study Group
IQR: interquartile range
OGTT: oral glucose tolerance test
OR: odds ratios
PG: plasma glucose
PG: plasma glucose
RCS: Restricted cubic spline
RERI: Relative excess risk due to interaction
SI: synergy index
SD: standard deviation
WCHC: Women and Children's Health Center.
