# Supplementary material for: Short Body Height and Pre-pregnancy Overweight for Increased Risk of Gestational Diabetes Mellitus: A Population-Based Cohort Study
Source: Front Endocrinol (Lausanne). 2018 Jun 26;9:349. doi: 10.3389/fendo.2018.00349 (PMC6028560; doi:10.3389/fendo.2018.00349)
Supplement: Supplementary file 1 [file Table_1.DOC]

Appendix table 1. Associations of short height with pre-pregnancy BMI and weight gain up to GCT per week.

|  | Height <158 cm | Height ≥158 cm | P value |
| --- | --- | --- | --- |
| **Univariable analysis** | | | |
| Weight gain per week, kg | 0.26±0.00 | 0.29±0.00 | <0.001 |
| Pre-pregnancy BMI, kg/m^2^ | 22.3±0.08 | 22.3±0.03 | 0.608 |
| **Multivariable analysis** | | | |
| Weight gain per week†, kg | 0.17±0.05 | 0.19±0.05 | <0.001 |
| Pre-pregnancy BMI‡, kg/m^2^ | 26.9±1.07 | 26.8±1.06 | 0.329 |

Abbreviations: BMI, body mass index; GCT, glucose challenge test; SBP/DBP, systolic/diastolic blood pressure.

†Adjusted for pre-pregnancy overweight, age, nationality, parity, SBP and DBP at GCT, DM history at first degree relative, education, habitual smoker and drinker, baby gender;

‡Adjusted for weight gain per week, age, nationality, parity, SBP and DBP at GCT, DM history at first degree relative, education, habitual smoker and drinker, baby gender.

Appendix table 2. Odds ratios (ORs) of short height and undue weight gain for the risk of gestational diabetes mellitus.

|  | N (%) | Odds ratio | 95%  confidence intervals | P  value |
| --- | --- | --- | --- | --- |
| **Independent models** | | | | |
| Univariable analysis† | | | | |
| Weight gain group, kg/wk | | | | |
| Undue weight gain: yes vs. no | 355(23.4%) | 0.99 | 0.87 to 1.12 | 0.842 |
| Multivariable analysis‡ | | | | |
| Height <158 vs. ≥158 cm | 126(11.2%) | 1.44 | 1.18 to 1.75 | <0.001 |
| Undue weight gain: yes vs. no | 274(24.4%) | 1.11 | 0.96 to 1.28 | 0.150 |
| **Independent models in the subgroups** | | | | |
| Univariable analysis among women with undue weight gain† | | | | |
| Height <158 vs. ≥158 cm | 24(6.8%) | 1.26 | 0.82 to 1.95 | 0.296 |
| Multivariable analysis among women with undue weight gain‡ | | | | |
| Height <158 vs. ≥158 cm | 22(8.10%) | 1.73 | 1.08 to 2.78 | 0.023 |
| Univariable analysis among women without undue weight gain† | | | | |
| Height <158 vs. ≥158 cm | 138(11.9%) | 1.33 | 1.11 to 1.61 | 0.003 |
| Multivariable analysis among women without undue weight gain‡ | | | | |
| Height <158 vs. ≥158 cm | 104(12.2%) | 1.39 | 1.11 to 1.72 | 0.004 |
| **Additive interaction models among the whole cohort** | | | | |
| Univariable analysis† | | | | |
| Height <158 cm & undue weight gain: yes | 24(1.6%) | 1.27 | 0.83 to 1.94 | 0.277 |
| Height <158 cm & undue weight gain: no | 138(9.1%) | 1.33 | 1.11 to 1.61 | 0.003 |
| Height ≥158 cm & undue weight gain: yes | 331(21.8%) | 1.00 | 0.88 to 1.14 | 0.957 |
| Height ≥158cm & undue weight gain: no | 1024(67.5%) | 1.00 | Reference |  |
| Multivariable analysis‡ | | | | |
| Height <158 cm & undue weight gain: yes | 22(2.0%) | 1.93 | 1.22 to 3.04 | 0.005 |
| Height <158 cm & undue weight gain: no | 104(9.3%) | 1.38 | 1.11 to 1.72 | 0.004 |
| Height ≥158 cm & undue weight gain: yes | 252(22.4%) | 1.09 | 0.94 to 1.27 | 0.262 |
| Height ≥158cm & undue weight gain: no | 746(66.4%) | 1.0 | Reference |  |

Abbreviations: BMI, body mass index; GCT, glucose challenge test; SBP/DBP, systolic/diastolic blood pressure.

Undue weight gain was defined as weight gain per week from the first antenatal care visit to GCT ≥ 75^th^ percentile or 0.37 kg.

†Not adjusted for any other variables;

‡The variables adjusted in the multivariable analysis included pre-pregnancy overweight, age, habitual smoker and drinker, Han-ethnicity, parity, SBP and DBP at GCT, education, family history of diabetes, baby gender, in addition to the variables listed in the model.

Appendix table 3. Addictive interaction between height <158 cm and undue weight gain for the risk of gestational diabetes mellitus.

| Measures of additive interaction | Estimate | 95%  confidence intervals |
| --- | --- | --- |
| **Univariable analysis**† | | |
| RERI | -0.07 | -0.67 to 0.52 |
| AP | -0.06 | -0.55 to 0.43 |
| SI | 0.79 | 0.09 to 6.74 |
| **Multivariable analysis**‡ | | |
| RERI | 0.46 | -0.53to 1.45 |
| AP | 0.24 | -0.19 to 0.66 |
| SI | 1.98 | 0.49 to 8.01 |

Abbreviations: AP, attributable proportion due to interaction; RERI, relative excess risk due to interaction; SI, synergy index; Significant RERI>0, AP>0 or SI>1 indicates a significant additive interaction;

Undue weight gain was defined as weight gain per week from the first antenatal care visit to GCT ≥ 75^th^ percentile or 0.37 kg.

†Not adjusted for any other variables;

‡The variables adjusted are the same as those in appendix table 2.

Appendix table 4. Odds ratios (ORs) of short height and old age for the risk of gestational diabetes mellitus.

|  | N (%) | Odds ratio | | | 95%  confidence intervals | | | | P  value |
| --- | --- | --- | --- | --- | --- | --- | --- | --- | --- |
| **Independent models** | | | | | | | | | |
| Univariable analysis† | | | | | | | | | |
| Age group, years | | | | | | | | | |
| Age ≥30 vs. <30 years | 538(35.5%) | 1.74 | 1.56-1.95 | | | | | | <0.001 |
| Multivariable analysis‡ | | | | | | | | | |
| Height < versus ≥158 cm | 126(11.2%) | 1.45 | | | 1.19-1.76 | | | | <0.001 |
| Age ≥30 vs. <30 years | 391(34.8%) | 1.52 | | | 1.33-1.74 | | | | <0.001 |
| **Independent models in the subgroups** | | | | | | | | | |
| Univariable analysis among women with age <30 years old† | | | | | | | | | |
| Height < versus ≥158 cm | 103(10.5%) | 1.40 | | 1.13-1.73 | | | | | 0.002 |
| Multivariable analysis among women with age <30 years old‡ | | | | | | | | | |
| Height < versus ≥158 cm | 81(11.1%) | 1.50 | | 1.18-1.92 | | | | | 0.001 |
| Univariable analysis among women with age ≥30 years old† | | | | | | | | | |
| Height < versus ≥158 cm | 59(11.0%) | 1.12 | | | | 0.84-1.49 | | | 0.457 |
| Multivariable analysis among women with age ≥30 years old‡ | | | | | | | | | |
| Height < versus ≥158 cm | 45(11.5%) | 1.36 | | | | | 0.97-1.90 | | 0.078 |
| **Additive interaction models among the whole cohort** | | | | | | | | | |
| Univariable analysis† | | | | | | | | | |
| Height <158 cm and age ≥30 years old | 59(3.9%) | 1.98 | | | | | | 1.50-2.62 | <0.001 |
| Height <158 cm and age <30 years old | 103(6.8%) | 1.40 | | | | | | 1.13-1.73 | 0.002 |
| Height ≥158 cm and age ≥30 years old | 479(31.6%) | 1.78 | | | | | | 1.58-2.00 | <0.001 |
| Height ≥158 cm and age <30 years old | 876(57.8%) | 1 | | | | | | Reference |  |
| Multivariable analysis‡ | | | | | | | | | |
| Height <158 cm and age <30 years old | 45(4.0%) | 2.06 () | | | | | | 1.48-2.86 | <0.001 |
| Height <158 cm and age ≥30 years old | 81(7.2%) | 1.51 () | | | | | | 1.18-1.92 | 0.001 |
| Height ≥158 cm and age <30 years old | 346(30.8%) | 1.54 () | | | | | | 1.33-1.77 | <0.001 |
| Height ≥158 cm and age ≥30 years old | 652(58.0%) | 1 | | | | | | Reference |  |

Abbreviations: BMI, body mass index; GCT, glucose challenge test; SBP/DBP, systolic/diastolic blood pressure.

†, Not adjusted for any other variables;

‡, The variables adjusted in the multivariable analysis included BMI, weight gain per week from first antenatal care visit to GCT, age, habitual smoker and drinker, Han-ethnicity, parity, SBP and DBP at GCT, education, family history of diabetes, baby gender, in addition to the variables listed in the model.

Appendix table 5. Addictive interaction between height <158 cm and old age for the risk of gestational diabetes mellitus.

| Measures of additive interaction | Estimate | 95%  confidence intervals |
| --- | --- | --- |
| **Univariable analysis**† | | |
| RERI | -0.19 | -0.83 to 0.45 |
| AP | -0.10 | -0.44 to 0.25 |
| SI | 0.84 | 0.45-1.55 |
| **Multivariable analysis**‡ | | |
| RERI | 0.01 | -0.75 to 0.78 |
| AP | 0.01 | -0.36 to 0.38 |
| SI | 1.01 | 0.49-2.09 |

Abbreviations: AP, attributable proportion due to interaction; RERI, relative excess risk due to interaction; SI, synergy index; Significant RERI>0, AP>0 or SI>1 indicates a significant additive interaction;

Old age was defined as age ≥30 years old.

†Not adjusted for any other variables;

‡The variables adjusted are the same as those in appendix table 4.

Appendix table 6. Sensitivity analysis with exclusion of 1209 women from the main analysis who registered for pregnancy after 14^th^ gestational week.

|  | N (%) | Odds ratio | 95%  confidence intervals | P  value |
| --- | --- | --- | --- | --- |
| **Independent models among the whole cohort** | | | | |
| Univariable analysis† | | | | |
| Height, cm | | | | |
| <158 versus ≥158 | 155(10.8%) | 1.34 | 1.12 to1.59 | 0.001 |
| BMI group, kg/m^2^ | | | | |
| ≥24 versus <24 | 456(31.6%) | 2.66 | 2.39 to 2.97 | <0.001 |
| Multivariable analysis‡ | | | | |
| Height, cm | | | | |
| <158 versus ≥158 | 120(11.2%) | 1.43 | 1.17 to 1.75 | <0.001 |
| BMI group, kg/m^2^ | | | | |
| ≥24 versus <24 | 495(46.2%) | 2.34 | 2.05 to 2.68 | <0.001 |
| **Additive interaction models among the women registration within 14^th^ gestational weeks** | | | | |
| Univariable analysis† | | | | |
| Height <158 cm & BMI ≥24 kg/m^2^ | 76(5.3%) | 3.70 | 2.85 to 4.79 | <0.001 |
| Height <158 cm & BMI <24 kg/m^2^ | 79(5.5%) | 1.26 | 0.99 to 1.60 | 0.061 |
| Height ≥158 cm & BMI ≥24 kg/m^2^ | 584(40.5%) | 2.63 | 2.34 to 2.95 | <0.001 |
| Height ≥158 cm & BMI <24 kg/m^2^ | 703(48.8%) | 1.0 | Reference |  |
| Multivariable analysis‡ | | | |  |
| Height <158 cm & BMI ≥24 kg/m^2^ | 60(5.6%) | 3.62 | 2.69 to 4.88 | <0.001 |
| Height <158 cm & BMI <24 kg/m^2^ | 60(5.6%) | 1.32 | 1.00 to 1.74 | 0.049 |
| Height ≥158 cm & BMI ≥24 kg/m^2^ | 435(40.6%) | 2.30 | 1.99 to 2.65 | <0.001 |
| Height ≥158 cm & BMI <24 kg/m^2^ | 516(48.2%) | 1.0 | Reference |  |

Abbreviations: BMI, body mass index; GCT, glucose challenge test; SBP/DBP, systolic/diastolic blood pressure.

†Not adjusted for any other variables;

‡The variables adjusted in the multivariable analysis included weight gain per week from first antenatal care visit to GCT, age, habitual smoker and drinker, Han-ethnicity, parity, SBP and DBP at GCT, education, family history of diabetes, baby gender, in addition to the variables listed in the model.

Appendix table 7. Additive interaction between height <158 cm and BMI ≥24 kg/m^2^ for the risk of gestational diabetes mellitus among women registration within 14^th^ gestational weeks.

| Measures of additive interaction | Estimate | 95% confidence intervals |
| --- | --- | --- |
| **Univariable analysis**† | | |
| RERI | 0.81 | -0.18 to1.80 |
| AP | 0.22 | 0.00 to 0.44 |
| SI | 1.43 | 0.96 to2.12 |
| **Multivariable analysis**‡ | | |
| RERI | 1.01 | -0.10 to 2.11 |
| AP | 0.28 | 0.04 to 0.51 |
| SI | 1.62 | 1.01 to 2.60 |

Abbreviations: BMI, body mass index; AP, attributable proportion due to interaction; RERI, relative excess risk due to interaction; SI, synergy index; Significant RERI>0, AP>0 or SI>1 indicates a significant additive interaction.

†Not adjusted for any other variables;

‡The variables adjusted are the same as those in appendix table 4.
